# Spatio-Temporal Urban Land Green Use Efficiency under Carbon Emission Constraints in the Yellow River Basin, China

**DOI:** 10.3390/ijerph191912700

**Published:** 2022-10-04

**Authors:** Hao Su, Shuo Yang

**Affiliations:** School of Public Administration, Xi’an University of Architecture and Technology, Xi’an 710055, China

**Keywords:** carbon peak, carbon neutralization, Super-SBM model, kernel density estimation, urban land green use efficiency, influencing factors

## Abstract

In the context of rapid urbanization and limited land amount, it is essential to scientifically evaluate the urban land green use efficiency (ULGUE) to promote regional sustainable development. Current studies are of great value for enriching the theoretical system and application research of ULGUE. Still, most of them only consider industrial pollution but ignore carbon emission as an essential environmental influencing indicator. This paper introduced carbon emissions into the input-output indicator system, measured ULGUE of 57 cities in the Yellow River Basin (YRB) over the 2004–2017 periods using the super-efficiency slacked-based measure (Super-SBM) model, analyzed its spatio-temporal patterns with the kernel density estimation (KDE) model and spatial autocorrelation model, and then identified the influencing factors with the Spatial Durbin model (SDM). As shown by the results, firstly, the ULGUE in the YRB over the 2004–2017 periods showed a trend of first decreasing and then increasing. Secondly, the ULGUE exhibited spatio-temporal imbalance characteristics across the YRB. Thirdly, ULGUE was the interaction of multiple indicators, and its influencing factors had spatial spillover effects. All in all, this paper is fundamental to the high-quality development of cities in the background of the Chinese policy of “carbon peak, carbon neutralization”.

## 1. Introduction

The land is the most fundamental natural resource and the material basis on which human beings depend for survival and development [1]. In addition, cities are areas of concentrated human activities, and their land sources are mainly from the conversion of neighborhood natural and agricultural land [2]. Since its reform and opening up, China’s cities have generated an enormous demand for land use as a product of rapid urbanization [3,4]. In 2020, the urbanization rate of China reached 63.9%, and the construction land area covered 58,355.3 km^2^. Under the dual constraints of China’s protection policy of farmland and ecological land, the urban construction land stock is limited, and the outward expansion of urban space is somehow hindered [5]. Over the past few years, the UnitedUN has been calling for the sustainable development of urban areas [6]. It is noteworthy that, the reasonable utilization of urban land resources is directly related to the rise and fall of cities. In the case of a limited amount of urban land, the efficient and intensive use of land resources has become the key to improving its utilization efficiency [7].

Early studies of urban land use efficiency (ULUE) are concentrated on the GDP output per unit of construction land area [8]. However, the continuous expansion of the city scale and the dramatic increase in the urban population, it has brought about some environmental problems, such as ecological deterioration, increased pollution, and insufficient greening infrastructure construction [9,10,11]. After considering undesirable outputs such as environmental pollution, the concept of urban land green use efficiency (ULGUE) has gradually been mentioned in scholarly studies [12,13]. In China’s primary national conditions of scarce resources per capita and fragile ecological environment, green development has become a strategic choice for development [14]. In fact, the concept of green development considering ecological boundaries has not only affected the traditional development concept of pursuing GDP growth, but also formed a new idea based on sustainable development and harmony between humans and nature as the goal [15].

Under the policy background of “carbon peak, carbon neutralization” in China, “green” has been given a new connotation in concept. China is the world’s largest carbon emitter, in which its energy carbon emission accounts for 28.8% of the world’s total energy carbon emissions [16,17]. Meanwhile, China has pledged to peak carbon emissions before 2030 and strive to achieve carbon neutrality before 2060 [18]. Urban areas contribute a major source of CO_2_ emissions, and produce over 78% of total carbon emissions on 2% of the global land share [19]. In 2022, the Chinese government officially issued the *Opinions on the Complete and Accurate Implementation of the New Development Concept for Carbon Neutrality* in 2022, which highlights “optimizing the regional layout of green and low-carbon development and building a new pattern of territorial space development and protection favorable to carbon peaking and carbon neutrality.” Under this circumstance, it is essential to consider the carbon emission factors in evaluating the land use efficiency level of cities, as industrial wastes and urban carbon emissions are equally significant in the evaluation of urban green development.

The Yellow River Basin (YRB), as an essential ecological barrier and a critical economic zone in China, spans four geomorphic units from west to east: The Tibetan Plateau, the Inner Mongolia Plateau, the Loess Plateau, and the Huanghuaihai Plain [20]. In 2019, China’s president Xi Jinping proposed a primary national strategy for ecological protection and high-quality development of the YRB at the *Symposium on Ecological Protection and High-Quality Development of the YRB* [21]. Indeed, the YRB consists of many resource-based cities with developed secondary industries and heavy energy consumption, which constitutes an essential source of carbon emissions and industrial pollution [22]. The scientific identification of the development pattern of cities in the YRB is significant for implementing national policies and promoting regional development. Overall, the study of ULGUE in the YRB cities is essential for the formulation of the relevant policy.

In this research, several questions should be answered: How was the level of ULGUE in YRB cities? How did it vary among cities? What were the influencing factors? Thus, this paper measured the ULGUE in the YRB over the 2004–2017 periods using the Super-efficiency SBM model, identified the spatio-temporal distribution characteristics using Kernel Density Estimation and the Moran’s index, and explored the influencing factors using the Spatial Durbin model. In brief, this study provides data support to improve the ULGUE and build low-carbon cities in the urbanization process of worldwide countries. The rest of the paper can be structured as follows: Section 2 is the review of the previous literature, and Section 3 is the theoretical analysis. Section 4 is an introduction to the materials and methods, and Section 5 presents the empirical analysis results of the ULGUE in the YRB. Besides, Section 6 is the discussion part and Section 7 draws the final conclusions.

## 2. Literature Review

The existing studies on land use efficiency can be explored from the following aspects. Specifically, the first is the method for measuring ULGUE from various perspectives. For example, Meng et al. measured the ULUE with the ratio of urban economic output and urban land area [23]. This method is often used to measure economic efficiency. Apart from that, Koroso et al. measured the efficiency of urban land use in terms of the population carrying capacity per unit built-up area [24]. Although, these measures are simple to calculate and easy to obtain, they cannot fully reflect the efficiency relationship between multiple factor inputs and multiple outputs in the urban land use process. Hence, the construction of a comprehensive multi-indicator evaluation system becomes the main research method for measuring ULUE, such as AHP, entropy method, and Bi-TOPSIS [25,26,27]. At the same time, there are some applications of production function measures in parametric methods, such as the Cobb-Douglas production function and Stochastic Frontier Analysis (SFA) models [28,29]. The Data Envelopment Analysis (DEA) method, as a non-parametric method, which can effectively evaluate decision units with many factor inputs and output indicators in a more objective way, gradually turns into the mainstream method for measuring ULGUE [30]. Besides, the Super-SBM model, which considers undesirable outputs, becomes scholars’ choices for efficiency measurement [31].

The second is the indicator selection for measuring ULGUE. From the perspective of inputs, Yang et al. and Zhang et al. selected indicators along relatively similar lines (i.e., land, capital, and labor) [32]. However, the specific choices for measuring these three inputs are somewhat divergent. The area of urban built-up areas or land under municipal districts for construction tends to be used as land inputs [33,34]. The number of persons employed in urban units or the total number of persons employed, including self-employed and private sectors, is often adopted to express labor input [35]. Beyond that, Ji et al. used the total population of a municipality instead of labor input [36]. The choice of indicators for an urban capital stock indicates capital inputs and is relatively consistent. In the measurement of the economic output of urban land, most studies have chosen municipal GDP or secondary and tertiary industry GDP [37]. The selection of green space coverage and green space area is more common in the desired environmental output [38]. Moreover, as a proxy for undesirable output, industrial wastewater, sulfur dioxide, and industrial soot emissions are the most frequently used indicators [39].

The third is the choice of influencing factors of ULGUE. At present, studies on the influencing factors of ULGUE can be divided into two categories. The first category probes into the relationship between individual influencing factors and ULGUE, mainly in terms of land finance, regional competition, urban form, urbanization, smart city construction, regional integration, etc. [40,41,42,43]. The second category is to analyze the integrated influences on urban land use efficiency. It has been proved that indicators such as economic development level, industrial structure upgrading, openness, population density, and land finance can significantly influence ULUE [44]. Meanwhile, scholars’ choices of influencing factors are limited by the availability of data.

In summary, the research results on ULGUE at home and abroad are relatively abundant, but it is essential to further explore some domains. From the perspective of indicator selection, the current studies do not form a unified input-output indicator system. Besides, the selection of different indicators leads to significant differences between the results. Most studies have taken “three industrial wastes” as undesirable outputs. Urban energy carbon emissions, as an essential factor affecting the ecological environment, should be included in the accounting of undesirable production. ULGUE, which takes account of industrial pollution and carbon emission indicators, is a new connotation guided by the concept of green development and should be adopted in subsequent studies. Also, although there are studies on ULUE or ULGUE with the YRB as the research area, most use the Tobit model to identify influencing factors, which merely consider the direct effects but ignore the possible indirect effects. A deeper excavation needs spatial econometric models for measurement.

## 3. Theoretical Analysis

### 3.1. Concept Definition

#### 3.1.1. Green Development

The concept of green development is accompanied by the deepening of human understanding of sustainable development [45]. In 1962, the idea of mutual checks and balances between environment and development was put forward by Rachel Carson in the book titled *Silent Spring***,** which was considered the germ of the birth of the concept of “green development” [46]. In the 1990s, British scholar David Pearce proposed the idea of a green economy and argued that economic development and environmental protection are in harmony and that integrating the concept of green development in economic and social development is a way to solve the contradiction between development and conservation [47]. In 2010, the United Nations Development Programme explained the meaning of a green economy as an economy that can improve human well-being and social equity while significantly reducing environmental risks and ecological scarcity. Beyond that, the 2015 United Nations Sustainable Development Summit adopted the *Transforming Our World—2030 Agenda for Sustainable Development*, which again stressed the importance of green development and ecological protection. What’s more, Organization for Economic Cooperation and Development (OECD) defined green development as the solution of balanced economic growth and development concerning the prevention of environmental degradation, loss of biodiversity, and unsustainable use of natural resources [48]. According to the World Bank, green development is an environmental-friendly way of economic growth, which aimed to efficiently use natural resources and minimize pollution emissions [49,50]. The definition of green development is not yet a unified perception in the academic field. In this paper, green development is defined as a development model with the mutual coordination of economic, social, and natural systems, which is mainly characterized by low consumption, low pollution, and increasing ecological capital.

#### 3.1.2. Urban Land

With the socio-economic development and the progress of industrial civilization, cities have gradually become the birthplace and center of modern society. For example, Weeks defined a city as a characteristic element that contains population density as well as social and economic organization and transforms the natural environment into a built environment [51]. Although urban land falls under the category of natural resources, it has its characteristics and includes not only natural resources consisting of topography, rocks, soil, water, biology, etc. but also the socio-economic resources that unite human labor in the process of land development and locational resources that are different in value. Meanwhile, scholars have various interpretations and definitions of the concept of urban land. As argued by Qadeer, urban land should not be divided simply by scope and area but should be defined according to its functional zoning [52]. As claimed by Deng et al., urban land is land within urban administrative districts, including all the land within the metropolitan area, suburban areas, and municipal counties of cities [53]. Tan et al. considered that urban land is the land within the built-up area, namely “the area within the urban administrative district that has been developed and constructed, and where municipal utilities and public facilities are available” [54]. In this paper, urban land refers to the urban built-up area, and the selected index is the built-up area of the municipal districts in the YRB.

#### 3.1.3. ULGUE

From the early studies, the domestic and foreign scholars initially limit the analysis of the concept of ULGUE to the economic level. For instance, Stull focused on defining ULGUE as the economic output generated per unit of urban land factor input from the perspective of land output intensity [55]. Apart from that, Chen not only introduced the concepts of structural efficiency of urban land allocation and the marginal efficiency of land use in China but also emphasized the re-examination of urban land use from the perspective of the market economy [56]. Studies combining land use efficiency with the concept of green development are at a later stage. Although Li et al. and Lu et al. considered environmentally undesirable outputs, they failed to specify the importance of “green” in the concept [57,58]. In 2018, Hu et al. linked green development and ULUE in the title for the first time [12]. In 2019, Liang et al. gave the first definition of ULGUE [13]. Since then, studies on ULGUE have begun to take shape. Especially, Zhao et al. defined ULGUE as the ratio of input factors to land use output of a land use system under certain production technology conditions [59]. As argued by Lu et al., the essence of ULGUE is to obtain the maximum land green economic output at the cost of the least possible land factor input and the minimum ecological loss [60]. According to Ji et al., the ULGUE manifests the comprehensive reflection of the degree of material cycle and energy exchange among the elements within the urban land use system and between the system as a whole and the external environment [36]. In this paper, ULGUE is defined as the social, economic, and ecological output capacity and level of all input elements of the urban land use system under the guidance of the green development concept.

### 3.2. Theoretical Compendium

#### 3.2.1. Land Intensive Use Theory

David Ricardo, the British economist, is the more academically recognized formal proponent of land-intensive use theory. He argued that the agricultural land’s intensive use denotes a form of agrarian production operation that yields a higher income on a certain amount of land through the investment of labor and other means of production as well as the use of advanced management and technological methods [61]. The theory of “smart growth” and “compact city” proposed by some scholars in Europe and America also emphasizes the intensive use of urban land [62]. In 1973, George Dantzig and Thomas Satty introduced the concept of a “compact city”, and proposed a new vision of efficient land use in terms of functional, structural, and scale compactness [63]. In 2000, the National Association of Planners in America proposed the “smart growth” model of urban development, which highlights the efficient use of existing land and shrinking urban boundaries by coordinating government spending with shrinking urban governance. Overall, the “urban growth boundary” under the smart growth concept has become an important measure to curb the blind expansion of urban land [63].

#### 3.2.2. Efficiency Theory

Efficiency, as the core issue in the production process, has been interpreted differently by different disciplines. Adam Smith proposed in *The Wealth of Nations* that the core of modern economics is the theory of efficiency, with the view of the division of labor efficiency and the theory of competitive efficiency as the essence [64]. Apart from that, neoclassical economics equates efficiency with allocative efficiency, and the research can be divided into two branches. Through the supply and demand partial equilibrium approach, Alfred Marshall investigated the theory of allocative efficiency that a perfectly competitive market can maximize efficiency, namely the optimal allocative efficiency [65]. The other is the Pareto allocative efficiency proposed by Pareto [66]. After reaching the Pareto allocative efficiency, no matter which forms of reconfiguration, there will not be at least one person’s efficiency that becomes better, while not making anyone’s efficiency worse. At the same time, the ratio of output to input can measure efficiency. Then, Farell defined a multi-input evaluation of firm efficiency, which shifted the study of efficiency from theory to empirical evidence [67]. In his view, efficiency consists of two components: technical efficiency and allocative efficiency. Technical efficiency is the ratio of the actual output of a production unit to the maximum output that can be achieved with constant inputs, whereas allocative efficiency is the ratio of the actual output of a production unit to the maximum output that can be achieved with output. What’s more, technical efficiency is the ratio of a production unit’s minimum cost to the unit’s actual cost, given a specific output. Therefore, ULGUE can be used to characterize the reasonable degree of urban land resource use.

#### 3.2.3. Location Theory

In 1826, Thunnen published his book *Isolated State*, which not only focused on the relationship between agricultural land use and land rent, but also opened up the theory of agricultural location [68]. Christaller, as the representative of neoclassical location theory, proposed the theory of central place in 1933, which provided a theoretical basis for the spatial organization and layout of cities [69]. Furthermore, Alonso published *Location and Land Use* in 1964, which elaborated the theory of competitive rents for land use and land prices within cities [70]. According to the classical location theory, Krugman introduced the latest research results of the new economic growth theory and world trade theory and constructed a unique spatial location theory based on the transformation and innovation of the traditional location theory [71]. The location theory, which is about the spatial allocation of resources and the spatial location choice of economic activities, plays an important role in the rational layout of the land. Meanwhile, the spatial interactions and associations among cities get closer, which prompts the coexistence of factor input and output systems in urban land use, and lays a theoretical foundation for the spatial interaction and regional differences of ULGUE.

### 3.3. Research Framework

**Figure 1:**
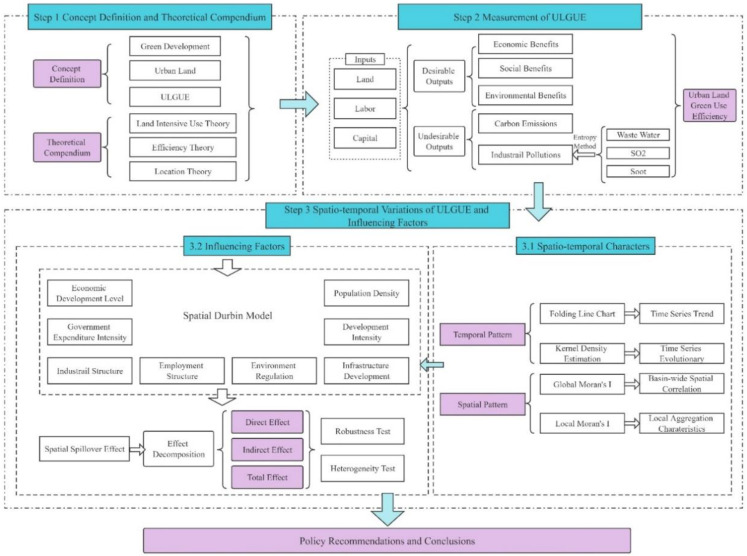
Research framework.

First, this paper defines the core concepts, which constitute the theoretical preparation of the study in combination with relevant theories. Based on the intensive land use and efficiency theory, this paper constructs an input-output index system to measure ULGUE in the YRB from 2004 to 2017. Further, based on spatial location theory, this paper investigates the characteristics of spatio-temporal divergence of efficiency among cities. Since there is a positive spatial autocorrelation of ULGUE, this paper uses a spatial econometric model to measure the influencing factors taking the spatial association into account, and conducts robustness tests and heterogeneity analysis. This paper then puts forward corresponding policy recommendations under the “theoretical analysis-empirical analysis” structure. The research framework is shown in Figure 1.

## 4. Materials and Methods

### 4.1. Study Area

**Figure 2:**
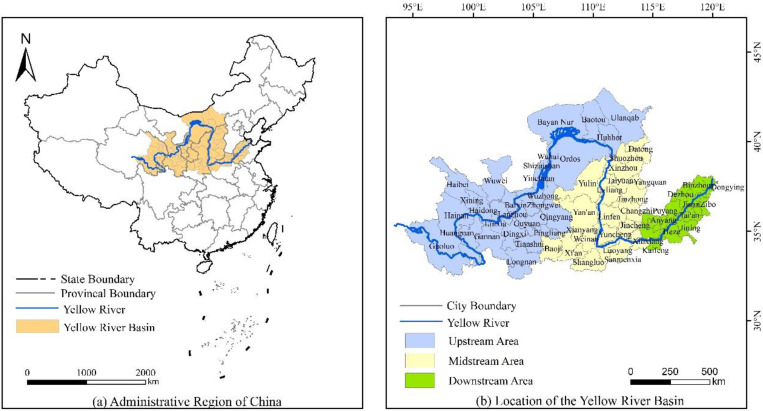
Location map of the Yellow River Basin, China.

The Yellow River Basin is located within 96°–119° E, 32°–42° N, and the total area of the basin is about 795,000 km^2^. The Yellow River originates from the northern foot of Bayankara Mountain in the Qinghai-Tibet Plateau, and is the second longest river in China, flowing through Qinghai, Sichuan, Gansu, Ningxia, Inner Mongolia, Shanxi, Shaanxi, Henan, Shandong 9 provinces and regions, the total length of 5464 km. The YRB is an important ecological security barrier in China, an important area of population activities and economic development. In the overall development of the country and the overall situation of socialist modernization, the YRB has a pivotal strategic position. Drawing on relevant research results and based on the natural YRB, considering the integrity of the geographical study unit, and the principle of a direct correlation between regional development and the Yellow River, the study area was defined as the eight provincial administrative regions of Qinghai, Gansu, Ningxia, Inner Mongolia, Shaanxi, Shanxi, Henan, and Shandong through which the Yellow River flows, with a total of 65 prefecture-level cities (lacking state and league data, 57 cities were finally selected) [21,72,73]. Taking the Hekou town in Inner Mongolia Autonomous Region and Taohuayu in Henan Province as the boundary, it is divided into the upper, middle, and lower reaches (Figure 2).

### 4.2. Super-SBM Model

The DEA method does not require the setting of a specific production function model, and can determine the weights of various input factors using objective optimization methods, avoiding artificial subjective factors, and can effectively evaluate decision units with multiple factor inputs and multiple output indicators in a more accurate manner, and has gradually become the mainstream method for measuring urban land use efficiency [74,75]. The SBM model constructed by Tone solves the problem of redundancy affecting the measurement values, which in turn overcomes the deficiency that the traditional model cannot compare efficiency values greater than 1 [76]. He further proposed the Super-SBM model, whose mathematical expression is as follows. This article used the iDEA 4.0 software (https://github.com/zsffgzs/iDEA, accessed on 9 August 2022) to perform the Super-SBM model.

(1) $$\begin{array}{c} \theta =\min\frac{1-\frac{1}{N}\sum_{n=1}^{N}\frac{S_{n}^{x}}{x_{k^{\prime n}}^{t^{\prime }}}}{1+\frac{1}{M+1\left(\sum_{m=1}^{M}\frac{S_{m}^{y}}{y_{k^{\prime }m}^{t^{\prime }}}+\sum_{i=1}^{I}\frac{S_{i}^{b}}{b_{k^{\prime }i}^{t^{\prime }}}\right)}}\\ s.t.\;\;x_{k^{\prime }n}^{t^{\prime }}=\overset{T}{\underset{t=1}{\sum }}\overset{K}{\underset{k=1}{\sum }}\lambda_{k}^{t}x_{kn}^{t}+S_{n}^{x},\;\;n=1,\ldots ,N\\ y_{k^{\prime }m}^{t^{\prime }}=\overset{T}{\underset{t=1}{\sum }}\overset{K}{\underset{k=1}{\sum }}\lambda_{k}^{t}y_{kn}^{t}-S_{m}^{x},\;\;n=1,\ldots ,M\\ b_{k^{\prime }i}^{t^{\prime }}=\overset{T}{\underset{t=1}{\sum }}\overset{K}{\underset{k=1}{\sum }}\lambda_{k}^{t}b_{ki}^{t}+S_{i}^{b},i=1,\ldots ,I\\ \lambda_{k}^{t}\geq 0,S_{n}^{x}\geq 0,\;S_{m}^{x}\geq 0,S_{i}^{b}\geq 0,\;k=1,\ldots ,K \end{array}$$

where *θ* is the urban land green use efficiency; *N*, *M*, and *I* are the input, desirable output, and undesirable output indicators, respectively; *x*, *y*, and *b* are the vectors of the three indicators; *S_n_^x^*, *S_m_^x^*, and *S_i_^b^* are the redundancies of the three indicators, respectively; $x_{k^{\prime }n}^{t^{\prime }}$, $y_{k^{\prime }m}^{t^{\prime }}$, and $b_{k^{\prime }i}^{t^{\prime }}\;$denotes the input-output values of *k′* production unit in period *t′*, *λ_k_^t^* is the weight coefficient of the decision unit. When *θ* ≥ 1, it means that the decision unit is at a higher efficiency level; when *θ* < 1, it means that with a certain efficiency loss, its input-output ratio has room for improvement.

### 4.3. Kernel Density Estimation

Kernel density estimation (KDE) is a nonparametric method capable of estimating the probability density of a random variable as a smooth peak function and modeling the distribution pattern of a random variable with a continuous probability distribution curve [77]. In this paper, we used KDE to identify the time-series evolutionary features of ULGUE in the YRB. The kernel density functions of ULGUE were estimated using Eviews 10.0 software (IHS Global INC., Irvine, CA, USA). The kernel density is calculated as follows:

(2) $$f\left(x\right)=\overset{n}{\underset{i=1}{\sum }}\frac{1}{h^{2}}k\frac{x-x_{i}}{h}$$

where *f(x)* refers to the estimate of the element’s kernel density at *x*, *h* refers to the bandwidth (search radius), *x*-*x_i_* is the distance from the *i*th element to the estimated element *x*, and *k (·)* denotes the weight function of the kernel.

### 4.4. Spatial Autocorrelation Model

#### 4.4.1. Global Moran’s Index

Spatial auto-correlation is an important indicator to test the correlated significance of the attribute value of an index with the attribute value of its adjacent space [78]. The Global Moran’s index reflects the correlation of attribute values of adjacent spatial units of the whole area. The absolute value of Moran’s index is close to 1, indicating a stronger spatial autocorrelation. The spatial distribution pattern of ULGUE from 2004–2017 was measured using Stata 15.1 software (StataCorp LLC., College Station, TX, USA). The Global Moran’s index can be calculated as follows:

(3) $$Global\;Moran^{\prime }s\;I=\frac{N\sum_{i}\sum_{ij}w_{ij}\left(x_{i}-\bar{x}\right)\left(x_{j}-\bar{x}\right)}{\left(\sum_{i}\sum_{ij}w_{ij}\right)\sum_{i}{\left(x_{i}-\bar{x}\right)}^{2}}$$

where *w_ij_*, *x_i_*, *x_j_*, *μ,* and *N* indicate the normalized weights, ULGUE in the *i*th pixel, ULGUE in the *j*th pixel, mean ULGUE of the study area, and the total number of pixels, respectively.

Moran’s index ranges from −1 to 1. When Moran’s index is greater than 0, there is a positive spatial correlation; when Moran’s index is less than 0, there is a negative spatial correlation. If the index is 0, then there is no spatial correlation.

#### 4.4.2. Local Moran’s Index

The Local Moran’s index (LISA) can effectively reflect the local correlation between the ULGUE of each city’s adjacent units within the area [79]. The LISA model was performed using the ArcGIS 10.8 software (Esri, Redlands, CA, USA). The calculation formula is as follows:

(4) $$Local\;Moran^{\prime }s\;I=\frac{\left(x_{i}-\bar{x}\right)\sum_{ij}w_{ij}\left(x_{j}-\bar{x}\right)}{\sum_{i}{\left(x_{i}-\bar{x}\right)}^{2}}$$

where the calculation parameters are the same as Global Moran’s index. LISA cluster map has 5 types of local spatial aggregation, namely High-High (H-H), Low-Low (L-L), Low-High (L-H), High-Low (H-L), and No Significant.

### 4.5. Spatial Durbin Model (SDM)

It has been confirmed that under the constraint of considering traditional industrial pollution, there was a certain spatial spillover effect of the influencing factors of ULGUE [37,40]. The traditional regression models do not consider spatial correlations [80]. In terms of model selection considering spatial correlation, the Spatial Autoregression Model (SAR) focuses on examining the spatial spillover effects and interdependence of the explanatory variables, the Spatial Error Model (SEM) reflects the spatial dependence caused by omitted variables, and the SDM model includes the above two spatial effects and considers the correlation between the spatial lagged term of the independent variable and the dependent variable [81,82]. Therefore, this paper applied the SDM model to measure the influencing factors of ULGUE and its spatial spillover effects in the YRB using the Stata 15.1 software. The spatial weight matrix *W* is calculated in the same way as the former Moran’s index. The preliminary formulated regression model equation is as follows:

(5) $$y_{it}=\rho \overset{n}{\underset{j=1}{\sum }}W_{ij}y_{it}+\beta x_{it}+\theta \overset{n}{\underset{j=1}{\sum }}W_{ij}x_{it}+\mu_{i}+\lambda_{t}+\varepsilon_{it}$$

where *y* is the ULGUE in the YRB, *i* denotes different years, *t* represents different regions, *W_it_* stands for the spatial weight matrix, *ρ* is the spatial regression coefficients of the explained variable, *θ* represents the spatial regression coefficients of explanatory variables, *μ_i_* and *λ_t_* represent spatial fixed effects and temporal fixed effects, respectively, and *ε* denotes the random error term.

### 4.6. Indicator Selection

#### 4.6.1. Indicators Measuring ULGUE

(1) Input indicators: Land, labor, and capital refer to three significant factors in production. With reference to the existing studies, the area of the built-up area was selected to characterize land input [83]. Beyond that, the total number of unit employees and private and self-employed workers in municipal districts at the end of the year was selected to characterize the labor input. Besides, the amount of urban capital stock characterizes the capital input. In this study, the perpetual inventory method was used to account for the amount of investment in the urban fixed assets with reference to Zhang et al., and the depreciation rate was set to be 9.6% [84]. Moreover, the fixed asset price index was used for each province to convert prices by the base period.
(2) Desirable output indicators: Based on the definition of ULGUE, this paper has set the expected output indexes from three perspectives: economic benefits, social benefits, and environmental benefits. To be specific, we used the GDP of secondary and tertiary industries in municipal districts as economic benefits and converted it into the constant price of 2004 using the GDP indices of different provinces [85,86]. Both urban employee salaries and total retail sales of social consumer goods, which reflect the social benefits, were treated to comparable numbers [87]. In addition, the area of parks and green spaces had been selected to evaluate the desirable output of environmental benefits [38].
(3) Undesirable output indicators: In this paper, the undesirable outputs were set to two aspects, namely the industrial pollutants and carbon emissions from residential and secondary, and tertiary industries. Normally, industrial sulfur dioxide emissions, industrial wastewater emissions, and industrial waste gas emissions were chosen to measure the undesirable outputs for efficiency studies [88]. In the case of a certain number of DMUs, too many output indicators of the DEA model will affect the accuracy of the results, and the units of all three industrial pollutants are different [60]. This study used the entropy method to synthesize the “three wastes” into an industrial pollution index, together with energy consumption and carbon emissions as two undesirable outputs. Furthermore, the carbon emission data used in this paper came from the research results of Shan et al., which have been widely applied in carbon emission accounting studies [19,89,90,91]. Notably, both industrial pollutants and carbon emissions are citywide statistical caliber. Therefore, this paper not only discounted the industrial pollution index by the proportion of the total industrial output value of the municipal district to the total industrial output value of the city but also discounted the carbon emissions by the proportion of the GDP of the municipal district to the GDP of the city. All the indicators and explanations are shown in Table 1.

#### 4.6.2. Influencing Factors of ULGUE

Combining the characteristics of YRB cities, eight relevant factors were selected to further investigate the influencing factors of ULGUE in the YRB. The information related to the variables is shown in Table 2. By measuring the city’s economic level, policy context, and development status, the following indicators were constructed. In addition, the GDP per capita was chosen to represent the level of economic development as an influencing factor for ULGUE [37]. The higher the GDP per capita of a city, the stronger its economic output capacity, but it may also harm the environment in the development process. Moreover, the industrial structure was measured by the share of secondary industry output in GDP [92]. Although the development of the secondary industry can rapidly improve the regional economy, it will influence the sustainable development of the ecological environment. Besides, the land-average year-end population was selected to measure the population density [93]. Higher population density causing a more developed tertiary sector may enhance land use efficiency but also generate more carbon emissions. We chose the ratio of public expenditure to GDP to measure government fiscal intensity [94]. The stronger the public expenditure of a city, the stronger the government’s ability to regulate the economy, but it may affect the free flow of resources. Meanwhile, the ratio of self-employed and private employees to total employment was chosen to measure the employment structure [36]. The private sector can increase market dynamics and facilitate capital flows. However, personal behavior is not easily regulated and may result in wasted resources. Beyond that, we used the road area per capita to evaluate the infrastructure development level [95]. Although convenient transportation will facilitate inter-regional exchanges, it may cause higher transportation costs in the resource allocation process. Then, we processed the entropy method to build an environmental regulation index by combing industrial solid waste utilization rate, domestic sewage treatment rate, and domestic waste harmless treatment rate [21]. Indeed, environmental regulations may reduce pollutant emissions, but they can increase the cost of technology adoption and create a capacity burden. Moreover, real estate development can boost the regional economy. Still, the conversion of large amounts of land to commercial use may prompt government departments to grant land for political performance, resulting in inefficient expansion of urban areas. Therefore, the real estate development investment completion amount per city area was selected to represent the development intensity.

### 4.7. Data Source

The socio-economic data were mainly obtained from the *China Urban Statistical Yearbook*, *China Urban and Rural Construction Statistical Yearbook*, EPS database, as well as the statistical yearbooks and statistical bulletins of various provinces and cities from 2005 to 2018. Carbon emission data was obtained from the CEADs (https://www.ceads.net, accessed on 1 August 2022). The GDP indices, CPI indices, and fixed asset price indices for each province were obtained from the National Bureau of Statistics of China. Vector data was obtained from the basic geographic database of the National Geographic Information Resources Catalogue Service of China (https://www.webmap.cn, accessed on 1 August 2022). The geographic distance weight matrix of cities in the YRB was generated and standardized based on the latitude and longitude locations of each city from Matlab 2019a (MathWorks, Natick, MA, USA). The inverse distance matrix for 57 cities was generated and standardized using Stata 15.1 software. Some of the missing values were supplemented by linear interpolation and the mean value method. The maps were visualized by ArcGIS 10.8 software.

## 5. Results

### 5.1. Time Series Variation Characteristics of ULGUE

**Figure 3:**
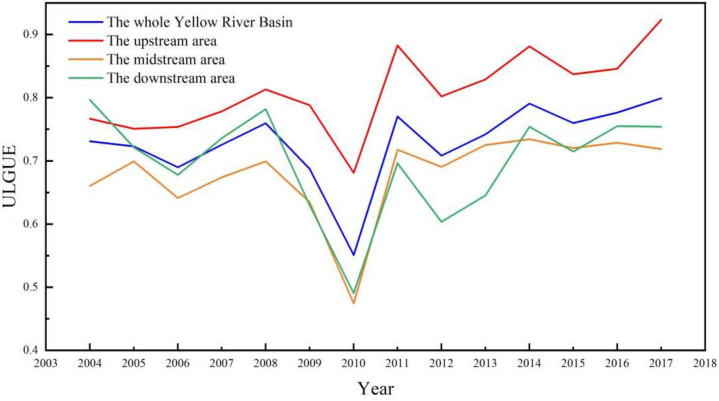
Average values of ULGUE of different areas in the YRB from 2004 to 2017.

We used the Super-SBM model with undesirable outputs to measure the ULGUE of 57 prefecture-level cities in the Yellow River basin from 2004–2017, and the results are as follows (Figure 3). According to the time-series analysis over the 2004–2017 periods, the changing pattern of the whole YRB, the upstream, the midstream, and the downstream remained consistent, with a fluctuating upward trend.

From 2004 to 2008, there were different degrees of improvement in ULGUE. Since China acceded to the WTO and the hosting of the Beijing Olympics, China’s socio-economy has been in a high growth stage. Thanks to the rapid economic growth, cities in the YRB accelerated the urbanization rate and the ULGUE gradually increased. Between 2008 and 2010, there was a large decrease in ULGUE. When the global financial crisis broke out in 2008, local governments of the YRB cities increased infrastructure construction to stimulate the economy, triggering over-exploitation of urban land resources and serious environmental pollution problems. Thus, ULGUE showed a decreasing trend in the period, which is consistent with the findings of Xue et al. [21]. From 2010 to 2017, ULGUE gradually increased in fluctuation and reached a peak. In 2011, the State Council issued the *National Main Functional Area Plan*, which divides the national land space into four functional areas according to development methods: optimizing development zones, key development zones, restricted development zones, and prohibited development zones. What’s more, adjustment and improvement of finance, industry, investment, land, population, environment, and other related plans and policies and regulations were required. This is consistent with the findings of Zhang et al. under the *National Main Functional Area Plan* [96]. Localities have actively promoted the intensive use of land resources and have paid attention to the protection of the ecological environment in the process of land use, thus promoting the ULGUE. Especially after 2014, China launched the *National New Type Urbanization Plan (2014–2020)*, which called for intensive and economical use of land resources, strengthened environmental protection, and boosted the improvement of ULGUE to some extent [21,97].

From a regional perspective, the time-series changes of the average ULGUE in the three different sub-regions were relatively consistent. From 2004 to 2017, the upstream area cities had the highest average ULGUE, while the ranking of the midstream area and the downstream area had fluctuating characteristics. From 2004 to 2008, the ULGUE in the downstream area was higher than that in the midstream. From 2008 to 2010, the ULGUE of both regions presented a sharp decrease. Between 2010 and 2013, the efficiency values of the two areas began to rise, and the mean value of the midstream region exceeded that of the downstream region. After 2014, the value of ULGUE in the downstream area was in the leading position, and the midstream area became the region with the lowest mean value of ULGUE in the YRB. According to the *National Sustainable Development Plan for Resource-based Cities (2013–2020)*, 38 resource-based cities in the YRB are mainly located in the midstream and downstream areas, with the largest number and most widely distributed in the midstream regions.

Notably, the development process of resource-based cities inevitably exerted a negative impact on the ecological environment, causing the problem of large industrial undesirable output emissions in the midstream area. Although the land use in the downstream area of the YRB was relatively intensive, the secondary industry was dominant most of the time due to the industrial structure, which also hindered the improvement of ULGUE. In contrast, the upstream region had a higher altitude, complex topography, and a fragile ecological environment. Thus, governments at all levels paid more attention to ecological environmental protection, and economic development was relatively slow compared to the middle and downstream regions. Besides, most of the cities belong to the low-income, low-emission, and high-efficiency types. Hence, the average ULGUE was higher.

**Figure 4:**
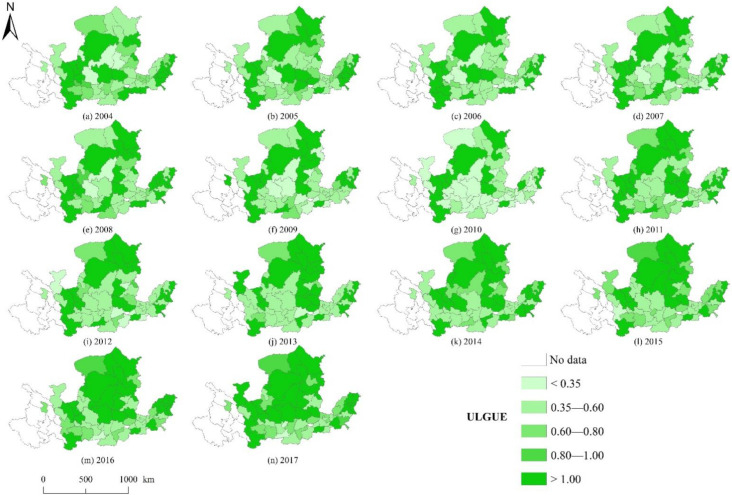
Spatial and temporal distribution patterns of ULGUE in the YRB from 2004 to 2017. (**a**–**n**) correspond to the years 2004 to 2017, respectively.

With reference to Xue et al., the ULGUE was divided into five levels according to efficiency less than 0.35, 0.35–0.60, 0.60–0.80, 0.80–1.00, and more than 1.00 (Figure 4) [21]. From 2004 to 2017, the cities with super-efficiency (efficiency value greater than 1) remained relatively stable, and the number presented a gradual increase since 2013. Specifically, Longnan, Dingxi, Guyuan, Wuzhong, Zhongwei, Baiyin, Erdos, Wuhai, and Shizuishan cities in the upstream area maintained stable DEA efficiency. By contrast, Xining, Lanzhou, and Yinchuan, as the capital cities of Qinghai Province, Gansu Province, and Ningxia Hui Autonomous Region, respectively, undertook the critical role of pulling economic development and industrial agglomeration. Therefore, the ULGUE was relatively inefficient under the constraint of undesirable outputs.

The cities in the upstream region mainly displayed the characteristics of the spaced distribution of high-efficiency cities with low environmental pollution and low development degree, while the capital cities had lower efficiencies. Among the midstream cities, there were fewer high-efficiency cities in 2004 and nine super-efficient cities in 2017, including Yulin, Zhengzhou, Lvliang, Yangquan, Yan’an, Changzhi, Linfen, Xinzhou, and Datong. In addition, the general land use in Shaanxi Province showed inefficient characteristics. Although Xi’an had reached the efficiency value of 1 during the 2006–2009 periods, with the biggest built-up area, the most extensive stock of fixed assets, and the largest number of employee inputs, it was difficult to achieve higher ULGUE at a lower carbon emission level with carbon emission constraints. In the downstream area, Zibo, Jinan, Dongying, Heze, and Binzhou cities achieved the DEA efficiency of ULGUE in 2017. The middle reaches of the Yellow River basin had the largest number of cities, and their efficiency values directly affected the level of urban land green use in the entire basin.

### 5.2. Evolutionary Features of ULGUE

**Figure 5:**
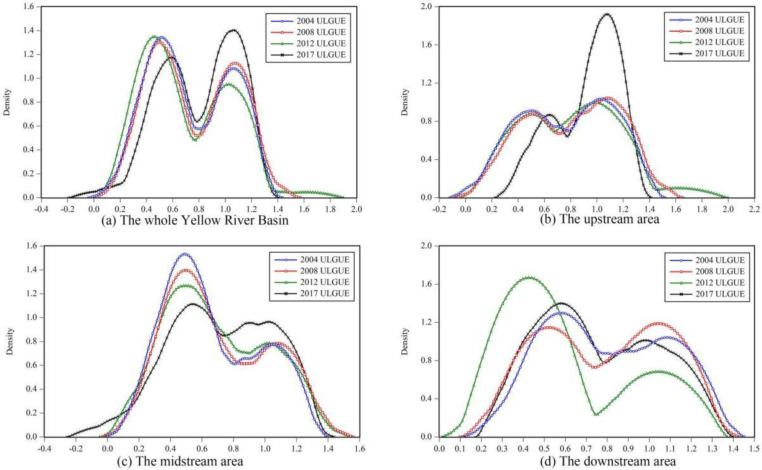
Evolution tendency of ULGUE in the YRB.

In this paper, the KDE model was used to measure the dynamic characteristics of their distribution for the whole, upstream, midstream, and downstream of the YRB in 2004, 2008, 2012, and 2017, respectively (Figure 5). Beyond that, the position of the main peak of the KDE curve can reflect the changing trend of the efficiency value, the height of the main peak can manifest the changing trend of the difference between efficiencies, and the number of waves can reflect the changing trend of the multi-polarization of the drawing efficiency value, and the shape of the main peak (broad peak and sharp peak) can embody the proportion of the high-value region and low-value region.

From a basin-wide perspective, the position of the main peak showed a trend of first leftward and then substantial rightward shift from 2004 to 2017, indicating that the ULGUE in the YRB experienced a trend from gradually decreasing to rapidly increasing during the study period. Among them, the main peak shifted slightly to the right from 2004 to 2008, then shifted significantly to the left from 2008 to 2012, and shifted even more significantly to the right from 2012 to 2017, presenting large volatility. The slight increase in the height of the main peak indicates that the difference between ULGUE of cities gradually decreases. Moreover, the simultaneous existence of the main and secondary peaks reveals that the bi-polar pattern of ULGUE has been present during the study period. The gradual steepening of the shape of the main wave peak indicates that the proportion of low-value areas of ULGUE gradually declines.

From a sub-regional perspective, the temporal evolutionary changes of ULGUE in the YRB, on the other hand, varied widely. The small difference in the height of the main peak and the secondary peak in the upstream area from 2004 to 2012 indicates that the changes in ULGUE are small. However, from 2012 to 2017, the main peak in the upstream was significantly elevated and shifted from a broad peak to a sharp peak, indicating that the difference of ULGUE among the upstream cities is gradually expanding and the proportion of the high-value area is gradually increasing. The main peak in the midstream region shifted slowly to the right, suggesting that the ULGUE displays a slowly increasing trend. Nevertheless, the height of the main peak decreased year by year, and there was a tendency for the sharp peak to turn into a broad peak, indicating that the difference between cities increased and the proportion of high-value areas declined. The main peak of the downstream region showed a “jump” change, shifting sharply right from 2004 to 2008, but shifting sharply left from 2008 to 2012, and shifting slightly right from 2012 to 2017. That is to say, the efficiency values have gone through a “rising-declining-rising” process. The width of the main peak of the KDE curves remained relatively stable, indicating that the variation of ULGUE among the downstream cities does not change to a great tent. The primary and secondary peaks of the KDE curves in the upstream, midstream, and downstream regions were all present, revealing that the differences in ULGUE are always present.

### 5.3. Spatial Distribution Characteristics of ULGUE

From 2004 to 2017, the results of Moran’s index of ULGUE in the YRB were negative for 4 years and positive for 11 years (Table 3). In addition, Moran’s index passed the significance level test in 4 years and failed the significance level test in 10 years. After 2013, the Global Moran’s index of ULGUE in the YRB increased year by year. That is to say, with the economic development of the YRB cities, the ULGUE gradually shows a more significant positive spatial dependence and spatial aggregation. Apart from that, one city’s urban land use efficiency will positively influence its neighboring cities’ ULGUE.

**Figure 6:**
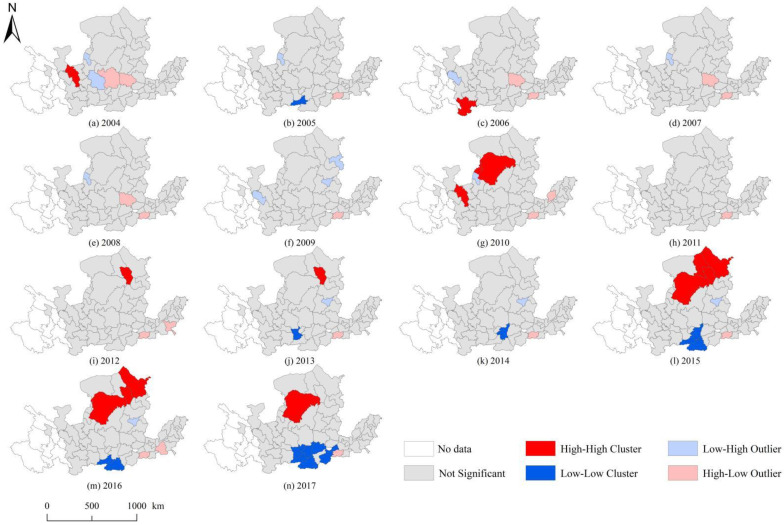
LISA cluster map of ULGUE in the YRB from 2004 to 2017. (**a**–**n**) correspond to the years 2004 to 2017, respectively.

Further, this paper reported the local Moran’s index, namely the “LISA” aggregation index of ULGUE (Figure 6). From 2004 to 2012, the high-high clustering, high-low clustering, low-high clustering, and low-low clustering alternated. After 2013, the high-low distribution characteristics of ULGUE were relatively stable. Among them, high-high aggregation appeared in Lanzhou, Longnan, Ordos, Hohhot, Baotou, and Ulanqab, while low-low aggregation emerged in Xi’an, Xianyang, Weinan, Shangluo, Tongchuan, Jincheng, and Jiaozuo. Beyond that, the ULGUE of cities within the Hohhot-Baotou-Ordos-Yulin Urban Agglomeration gradually reached DEA efficiency. In contrast, the ULGUE of the Guanzhong Plain Urban Agglomeration displayed a significant low-low aggregation feature.

### 5.4. Regression Results of the SDM Model

The independent variables were logarithmized to enhance the smoothness of the data, reduce covariance, and mitigate heteroskedasticity. Prior to conducting the regression analysis, a test for multicollinearity was first performed, and the mean VIF was 3.06, much less than 10, indicating that there is no multicollinearity among the selected variables. Furthermore, unit root tests and cointegration tests were carried out. There was a long-term equilibrium relationship between ULGUE and the selected variables, which can be subjected to panel regression analysis.

In the LM test, Moran’s index of the SEM model was significant at the 1% level, and Moran’s index of the Spatial Lag Model (SLM) was also significant at the 1% to 10% level. Under the premise that both models were applicable, the SDM model that combined both could be chosen for empirical analysis. Then, the Hausman test was conducted for the choice of random or fixed effects and the *p*-value was less than 0.1. Therefore, the SDM model should choose the fixed effects for the regression. The LR test and Wald test both passed the significance test at the 1% level, implying that the SDM model will not degenerate into the SAR model and SEM model in the regression analysis. According to the results of LR tests for time-fixed, spatial-fixed, or time-spatial-fixed effects, the time-spatial-fixed effect would be suitable for the SDM model (Table 4).

The regression results of the SDM model are shown in Table 5. According to the results of the LR tests for time fixed effects, spatial fixed effects, and time-spatial fixed effects, the analysis of SDM regressions should be selected with time-spatial dual fixed effects. The regression coefficient of lnpgdp, which was used to measure the regional economic development level, was positive and passed the 1% significance test, indicating that the improvement of the city’s local economic development level is conducive to the advancement of ULGUE, which is consistent with the findings of Chen et al. and Zhu et al. [75,98]. Apart from that, the urban area is the core for developing secondary and tertiary industries, which requires the intensification of land resource utilization. In addition, the level of economic development of a city is a concentrated manifestation of the scale economy, and the higher the per capita GDP of a city, the more it indicates that high-quality development can be achieved with limited land resources.

The regression coefficient of employment structure was −0.071, which negatively affected ULGUE at a 5% significant level, which is consistent with the findings of Ji et al. [36]. Additionally, flexible private and individual employment is conducive to reducing transaction costs in the resource allocation process and improving resource utilization efficiency. Still, the congestion costs in the process of resource agglomeration reduced the output efficiency of input resources for urban development. Particularly, some cities had been attracting investment at the cost of cheap land for a long time, which decreased the volume ratio of local industrial land. In China’s policy of intensive energy use, state-owned enterprises and units often have targets for reducing energy consumption per unit of GDP. In contrast, individual private enterprises are not easily constrained.

The coefficient of environmental regulation was −0.105 and was significant at a 5% statistical level, indicating that the level of environmental regulation harms ULGUE, and there is no “Porter’s hypothesis” for ULGUE in the YRB, which is consistent with the findings of Hao et al., Wang et al., and Shuai et al. [99,100,101]. Meanwhile, when a region’s economic development level is low, there is a small possibility of improving environmental pollution, achieving technological innovation, and meanwhile increasing output value. The innovation compensation obtained by each production sector through environmental regulation is less than the increased production cost. Accordingly, the increase in the environmental regulation level will reduce the ULGUE instead.

The coefficient of real estate development intensity was −0.028, which was significant at the 10% statistical level, indicating that high-intensity real estate development will suppress the regional ULGUE. In the “land economy” of China, government departments have promoted economic growth to a certain extent by granting land and bringing in real estate developers. Still, the use of land resources has shifted to commercial and residential land. What’s more, the restricted land use pattern is not conducive to developing urban ecological functions.

The spatial spillover effects of the SDM model were measured, namely direct effect, indirect effect, and total effect decomposition (Table 6). Except for government financial expenditure, the positive and negative characteristics and significance of the regression coefficients for the direct effects remained consistent with the main regression results. Additionally, the direct effect of government financial expenditure strength was −0.057, which passed the 10% significance level test, indicating that the degree of local urban government intervention inhibits the improvement of local ULGUE. It is consistent with the findings of Tu et al. and Liu et al. that government intervention exerts a negative effect on urban land use efficiency [19,102]. The excessive governmental intervention will crowd out the participation of more competitive and active market players in urban land development. In this section, the indirect effects of the influencing factors are mainly analyzed.

The indirect effect of governmental expenditure intensity was significantly positive at the 1% level, indicating that the increased fiscal expenditure of local government positively affects the ULGUE in neighboring cities. Local governments have multiple game relationships in the face of environmental pollution and environmental management problems in socio-economic development. Under the pressure of economic growth and political promotion, local governments’ interventions in socio-economic development are spatially competitive. Apart from that, local city governments’ policy measures and strategic behaviors in land development and utilization, investment in urban infrastructure, and industrial layout will also have positive spatial transmission to the neighboring cities through demonstration, imitation, learning, and competitive effects.

The indirect effect coefficient of the industrial structure was −0.738, which passed the significance test at the 10% level, indicating that the degree of second industry proportion of a city has a negative spillover effect on the ULGUE of its neighboring cities. In comparison to the tertiary sector, a higher share of the secondary industry will take up more land, consume more energy, and cause more industrial pollution and carbon emissions [103]. The spatial aggregation and industrial development of resource-based cities in the YRB are distinctive, and the spatial spillover effect influences the ULGUE of the neighboring cities.

The indirect effect coefficient of environmental regulation was 0.868, which passed the significance test at the 5% level. The increase in environmental regulation intensity in local cities is conducive to promoting the improvement of ULGUE in neighboring cities. Beyond that, industries of high energy consumption, high pollution, and high emission industries are forced to improve pollution emission treatment technology in the production process and raise awareness of green production. The cities have similar industrial structure characteristics to the YRB, which is densely populated with resource-based cities. In the context of China’s policy of intensive energy use, industrial pollution remediation, and haze remediation, there is competition for political achievements in each city. Moreover, the improvement of environmental regulations in one city will trigger the surrounding cities to follow suit, which in turn propels the improvement of ULGUE.

### 5.5. Robustness Test

#### 5.5.1. Independent Variable Replacement

In this study, robustness tests were performed by replacing the independent variables. Specially, we use the share of the tertiary sector in GDP to represent the industrial structure and the centralized sewage treatment rate to represent environmental regulation and again run the regression referring to Xue et al.’s study [21]. The results of variables remained stable (Table 7). According to the regression results, the coefficient of the indirect effect of industrial structure is 0.757, which passes the significance test at the 5% level. Besides, the coefficient changed from “negative significant” in the main regression to “positive significant”. The direct effect of environmental regulation is −0.114, which is significant at the 5% level, whereas the indirect effect coefficient is 0.975, which is significant at the 1% level. The coefficients and significance levels of the remaining variables also remain consistent, indicating that the findings of this paper are somewhat robust.

#### 5.5.2. Replacement of the Spatial Weight Matrix

To test the robustness of the findings, this paper used the inverse distance matrix to replace the geographic distance matrix for the regression analysis of the SDM model. As shown in Table 8, after replacing the spatial weight matrix, the signs and significances of the direct and indirect effects of the influencing factors of ULGUE remained basically the same, indicating that the conclusions of this paper are robust.

### 5.6. Heterogeneity Test

The YRB spans eight provinces, and the 57 sample cities selected in this paper have variability in geographic location, economic development level, resource endowment, and government management characteristics [104]. In this section, we classified cities in the YRB into upstream, midstream, and downstream cities to further discuss the heterogeneity of factors influencing ULGUE. In the regression results of the SDM model (Table 9), government expenditure intensity, employment structure, and environmental regulation of upstream cities could significantly negatively affect ULGUE. Apart from that, the economic development level positively affected the ULGUE of midstream cities, while employment structure negatively influenced the ULGUE at a 10% level. In addition, the ULGUE of downstream cities was positively influenced by the economic development level at the 10% level, and it was significantly negatively affected by environmental regulation and the real estate development intensity. As for the analysis of the estate development, a lot of capital flows into the real estate industry, which is unfavorable to the flexible allocation of resource factors. Besides, the real estate industry has formed a rigid trap in the development process, which will hinder the development of sustainable cities [105].

Regarding the indirect effects, spatial spillover effects were mainly manifested in midstream and downstream cities. To be specific, the economic development levels of midstream and downstream cities significantly negatively affected the surrounding cities’ ULGUE. Unlike the sparse distribution of upstream cities, the midstream and downstream cities are more closely connected due to geographical and economic factors. When the scale of a city far exceeds that of its neighboring cities, it will form a siphon effect, attracting a large amount of labor, capital, and new industries and forming an unbalanced development structure [106]. Furthermore, the industrial structure in midstream cities significantly and negatively affected the efficiency of the surroundings. In the midstream, where resource-based cities are densely distributed, the second industry agglomeration of cities negatively influences the surrounding cities [107].

## 6. Discussion

### 6.1. ULGUE Variations in the YRB

The YRB is the main supply base of energy resources in China and an ecological corridor connecting the Qinghai-Tibet Plateau, Loess Plateau, and North China Plain. Since the ecological protection and high-quality development of the YRB have been promoted as a national strategy, a lot of scholars have paid extensive attention to the sustainable development of this region [20,108,109]. In general, scholars conducted research on ULGUE in the YRB from two perspectives: industrial pollutants as the undesirable outputs and carbon emissions as the undesirable outputs. Since these two research perspectives are different, their’ conclusions have common points and differences. Taking industrial pollutants as the undesirable outputs, Xue et al. calculated the ULGUE in the YRB and found that the efficiency values in the whole YRB, upstream, middle, and downstream, show a decreasing and then increasing pattern (the average upstream efficiency > downstream efficiency > midstream efficiency) [21]. These are consistent with the findings of this paper. Besides, Li et al. selected the treatment rate of industrial waste gas, wastewater, and soot as the desirable outputs in the SBM model, and measured the ULGUE in the YRB as well as its spatial distribution pattern [87]. According to the LISA aggregation results, the low-low aggregation area was in the Guanzhong Plain and the Loess Hills. In contrast, the high-high aggregation area was in the “jizi” bends of the Yellow River and the downstream area. These can also justify the results of this paper. Apart from that, Wu et al. measured the effect of urban form on urban land use efficiency in the YRB under the carbon emission constraint [110]. Unlike the municipal district perspective in this paper, their research perspective was the whole city in the administrative sense, leading to the difference in the conclusion. Taking carbon emissions as the undesirable outputs, Liu et al. examined urban land use efficiency at the national level and found that cities at the national level are collectively inefficient under the carbon emissions constraint [19]. Increasing carbon emission is an essential issue in urban green development, which explains the inefficiency of some cities with higher levels of economic growth after the introduction of carbon emissions as an undesirable output in this paper. This is also consistent with the findings of Chen et al. [111].

### 6.2. Paradigm of Low Carbon Development

With economic globalization, substantial carbon emissions and industrial pollution have created an enormous obstacle to sustainable development [112]. At the same time, countries are facing different costs of carbon emission reduction, such as direct economic and social costs, under the constraints of their national conditions [113]. In this context, learning from the practical experiences of countries and the research results of scholars can help build a low-carbon paradigm and provide ideas for urban governance.

From the perspective of natural factors, improper soil management can cause a reduction in the carbon sink capacity of the land [114]. The global terrestrial carbon sink capacity has significantly improved, and terrestrial ecosystems can absorb about 16% of carbon dioxide emissions from fossil fuels. Also, ocean carbon sinks have become an essential source of net carbon sinks [115]. Human activities are putting pressure on the global carbon cycle. From a technological perspective, scholars have studied the path to carbon neutrality from the viewpoint of clean energy, carbon sequestration, and infrastructure development [116,117,118]. From a policy perspective, Du et al. demonstrated that trade barriers increase inequality in resource allocation, and increased tariffs imply increased costs of carbon emission reduction and increased air pollution and mortality [119]. Yang et al. found that rational use of Nitrogen fertilizers, optimizing the energy mix of buildings, and using clean energy can reduce carbon emissions [120]. The above three perspectives can provide pathway support for global carbon management.

The construction of a low-carbon city is a multidisciplinary and jointly guided vision of the future city, whose definition and criteria are dynamic. Moreover, low-carbon development requires bottom-up low-carbon behaviors, up-bottom policy guidance, and technical support that constantly running through it. At present, cities around the world are exploring new models of low-carbon development [121]. In order to reduce the impact of climate change, the city of Boston in the United States has announced the goal to achieve carbon neutrality by 2050. More specifically, the city has developed detailed climate change mitigation strategies and action steps in the implementation plan in three areas: buildings, transportation, and other emission reduction measures. In 2012, Copenhagen of Denmark proposed the world’s first carbon-neutral capital city plan, which sets out specific goals and initiatives in the areas of energy consumption, energy production, green transportation, and urban management. The plan sets out specific targets and ambitions. At the same time, Vancouver city’s government has proposed to reduce fossil fuel combustion in vehicles and buildings and achieve carbon neutrality by 2050 by encouraging low-carbon transportation, developing low-carbon buildings, and increasing carbon sinks to mitigate climate change. Having been actively pursuing carbon reduction policies, China identified three batches of pilot cities in 2010, 2012, and 2017, respectively, which were proven to be significant for reducing emissions with target and neighboring cities [122]. Apart from that, small towns in China have also carried out low-carbon town planning based on the idyllic city theory. Furthermore, countries worldwide are actively exploring and making unremitting efforts to build low-carbon cities with regional characteristics.

### 6.3. Policy Implications for Improving ULGUE

(1) Controlling the land supply amount, adhering to the urban development boundary, and improving ULGUE within the city’s built-up areas. The government should consider factors such as the carrying capacity of resources and the environment and the suitability of land space development. In addition, the economic development patterns should be based on local conditions. Meanwhile, urban development mode driven by the expansion of construction land together with the “pie” type of urban development should be strictly forbidden. It is also essential to promote the structural reform on the supply side of urban land and change from the development mode of traditional urban space expansion land to the optimization mode of urban land space layout. Moreover, upstream and midstream cities need to enhance the construction of green space facilities to increase the expected output of land. Besides, the downstream cities should promote the development of the real economy and reduce the real estate economic bubble with consideration of the economic development level.
(2) Accelerating the green manufacturing industry, with the green sustainable development concept as the guidance. Industrial structure optimization and moderate intensification are encouraged to provide the ecological foundation for the green sustainable and healthy development of cities and livable cities. The long-standing development mode in the YRB has resulted in a large proportion of traditional manufacturing and resource-based industries in the industrial structure. At the same time, there is a low proportion of high-tech, advanced manufacturing, and modern service industries as well as a severe phenomenon of industrial homogeneity. It is necessary to rely on industrial transfer and industrial structure upgrading to promote the advanced manufacturing industry. With energy conservation and emission reduction as the policy guidance, the government should not only accelerate the implementation of peak planning and action plans for major cities and energy carbon-emitting industries, but also incorporate the implementation of carbon-emission control and air pollutant emission reduction into the central environmental protection inspectors, local party, and government ecological and environmental leadership of the responsibility audit system.
(3) Actively promoting the synergistic development among cities and city agglomerations in the YRB in the context of regional integrated development. Departments should take the core cities and metropolitan areas as the entry point, pull the surrounding cities with their comprehensive advantages, further link the surrounding node cities to form a radiation circle, and gradually drive the development of the small and medium-sized cities. Since there is an increasing spatial spillover effect of ULGUE, the governmental departments should actively review the spatial interaction between local cities and neighboring cities and strengthen the role of linkage control between cities in land urbanization. Apart from that, the ULGUEs of the Lanzhou-Xining Urban Agglomeration and Hohhot-Baotou-Ordos-Yulin Urban Agglomeration is relatively high, while the Guanzhong Plain Urban Agglomeration is at a lower efficiency level. It is of great significance to use the positive spillover effect of government financial support, industrial restructuring, and infrastructure construction to promote the positive transmission of ULGUE in cities.

### 6.4. Limitations and Future Prospects

This paper may have the following research shortcomings. First of all, due to the lack of accurate and complete energy consumption data for prefecture-level cities, this paper used the carbon emission dataset based on the nighttime light inversion provided by Chen et al. with a time cut-off of 2017 [91]. Secondly, this paper mainly considered carbon emissions, but ignored carbon sequestration in the model construction. For instance, there was a lack of data on land use types in the municipal area to obtain the precise area of trees, grasslands, and waters in consecutive years. Thirdly, industrial pollution and carbon emissions in municipal districts were converted through the GDP share, which generated some errors. However, after considering the dual environmental constraints of carbon emissions and industrial pollution, this paper is of great significance for scientifically and accurately measuring the ULGUE in YRB cities. In future research, we will use carbon emission data with higher precision and updated years, combine it with land use data, and investigate ULGUE at the national scale as well as the mechanism of specific policies on it.

## 7. Conclusions

The increasing urbanization rates have made cities the regional centers of economy, population, and resources. For cities worldwide, it is a common problem to balance economic, social, and ecological benefits and achieve high efficiency with the currently limited amount of land is a common problem. In addition, the resident’s livelihood and the production of industry all produce large amounts of greenhouse gas emissions and environmental pollution, which will affect the sustainable development of the YRB. In this paper, an improved input-output indicator system was developed to measure regional ULGUE, which can also be used in other regional and national studies. From the perspective of urban carbon emissions as undesirable outputs, this paper measured the ULGUE of 57 prefecture-level cities in the YRB over the 2004–2017 periods using the Super-SBM model, analyzed their spatio-temporal divergence characteristics with the KDE model and spatial autocorrelation, and explored the influencing factors and spatial spillover effects. The main conclusions are as follows.

(1) The temporal change patterns of ULGUE in the Yellow River Basin exhibited a “U”-shaped feature. Namely, it declined first and then increased with the development of the cities. The ULGUE in the YRB decreased from 2004 to 2010, while rising to a higher level from 2010 to 2017, which indicates the possible existence of an environmental Kuznets curve in the development of the urban economy. The average efficiency in the upstream area was the highest, followed by the downstream area and the midstream area (lowest). Although most upstream cities have a pattern of “low development level and low emission” pattern, the downstream cities have an agglomeration characteristic of “high development level and high emission”. There are many resource-based cities in the midstream, with significant differences among cities. This provides empirical support for the recognition of ULGUE in multiple types of cities.
(2) ULGUE in the upstream, midstream, and downstream areas presented distinctive spatial non-equilibrium characteristics. Especially, the bi-polar pattern of ULGUE had been present during the study period. The spatial autocorrelation of ULGUE during 2004–2013 was insignificant, but a significant positive spatial correlation increased yearly after 2013, indicating that the positive inter-regional synergy feature is becoming more decisive annually. Meanwhile, the natural resource endowments can also influence the spatial distribution of ULGUE. What’s more, the Local Moran’s index displayed a “north high and south low” distribution. The Inner Mongolia Plateau and Loess Plateau regions were the high-value areas for ULGUE, while the Guanzhong Plain was a low-value aggregation area. That is to say, areas with low topographic relief and low elevation are easy for urban construction, but not easy for intensive use of land resources. In this regard, more scientific urban planning and policy support are needed.
(3) Under the spatial spillover effect, the influencing factors of ULGUE showed a complex mechanism. From the perspective of a single city, the regional economic development significantly improved the ULGUE, while the fiscal expenditure intensity, employment structure, environmental regulation, and development intensity significantly negatively affected the ULGUE. Considering the decomposition of direct effects, indirect effects, and total effects, the fiscal expenditure intensity, environmental regulation, and industrial structure (high percentage of tertiary industry) had a significant positive spatial spillover effect. Apart from that, the total effects of fiscal expenditure intensity, environmental regulation, and industrial structure were all positively significant. Besides, the midstream and downstream were more prone to economic siphoning impacts, resulting in inefficient ULGUE of the neighboring cities. In short, reasonable policy regulations are needed to adjust the ULGUE of a region.

## Figures and Tables

**Table 1:** Input-output index table of Super-SBM model.

Indicator	Variable Type	Variable Explanation	Unit
Input	Land	Urban built-up area	km^2^
	Labor	Total number of urban employees	10 thousand person
	Capital	Urban capital stock	billion CNY
Desirable Output	Economic benefits	Secondary and tertiary industry GDP in municipal districts	billion CNY
	Social benefits	Total retail sales of social consumer goods	10 thousand CNY
		Urban employee salary	CNY
	Environmental benefits	Area of parks and green spaces	hm^2^
Undesirable Output	Industrial Pollution	Composite index synthesized by the entropy method including industrial wastewater, SO_2_, and soot emissions	/
	Carbon Emission	Carbon emissions from urban energy consumption	million ton

**Table 2:** Influencing indicators of the ULGUE.

Variable Name	Variable Content	Variable Explanation	Unit
pgdp	Economic development level	Gross Domestic Product per capita	CNY/person
is	Industrial structure	Secondary industry output value/total GDP	%
pd	Population density	Year-end population/area of the municipal district	Person/km^2^
ge	Governmental expenditure intensity	Public finance expenditure/total GDP	%
es	Employment structure	Number of self-employed and private employees/total number of employees	%
road	Infrastructure Development	Road area per capita	m^2^/person
er	Environmental regulation	A composite index of industrial solid waste utilization rate, domestic sewage treatment rate, and domestic waste harmless treatment rate generated by the entropy method	/
eip	Development intensity	Real estate development investment completion amount/city area	10 thousand CNY/km^2^

**Table 3:** Global Moran’s I values of YRB from 2004 to 2017.

Year	Moran’s I	Z-Score	*p*-Value	Year	Moran’s I	Z-Score	*p*-Value
2004	−0.001	0.821	0.411	2011	0.006	1.127	0.260
2005	0.002	0.945	0.345	2012	0.003	1.003	0.316
2006	−0.023	−0.223	0.823	2013	0.024	2.027	0.043
2007	−0.023	−0.237	0.812	2014	0.030	1.729	0.084
2008	−0.014	0.175	0.861	2015	0.040	2.749	0.006
2009	0.005	1.097	0.272	2016	0.055	3.426	0.001
2010	0.009	1.281	0.200	2017	0.064	3.882	0.000

**Table 4:** Test results of spatial panel model selection.

Test	Test Statistics	*p*-Value
LM-Lag	3.018	0.082
Robust LM-Lag	7.691	0.006
LM-Err	9.185	0.000
Robust LM-Err	6.290	0.012
Wald test for SAR	34.160	0.000
Wald test for SEM	36.520	0.000
LR test for both and spatial fixed	64.680	0.000
LR test for both and time fixed	589.940	0.000
LR-SDM-SAR	33.320	0.000
LR-SDM-SEM	35.570	0.000
Hausman	13.500	0.096

**Table 5:** Regression results of the SDM model.

Type	(1) SDM	(2) SDM	(3) SDM
	(Spatial Fixed)	(Time Fixed)	(Time-Spatial Fixed)
Main			
lnpgdp	0.221 *** (4.65)	0.211 *** (8.82)	0.227 *** (4.92)
lnge	−0.074 ** (−2.87)	0.084 *** (3.82)	−0.047 (−1.88)
lnis	0.033 (0.57)	−0.281 *** (−8.15)	0.014 (0.25)
lnes	−0.073 ** (−3.14)	−0.142 *** (−5.86)	−0.071 ** (−3.09)
lnroad	−0.019 (−0.82)	0.024 (1.20)	−0.018 (−0.80)
lner	−0.126 ** (−3.06)	−0.178 *** (−3.97)	−0.105 ** (−2.60)
lnpd	−0.016 (−0.38)	−0.063 ** (−2.90)	−0.011 (−0.26)
lneip	−0.023 (−1.82)	−0.019 (−1.27)	−0.028 * (−2.27)
Spatial rho	0.431 *** (4.46)	−1.494 *** (−6.10)	−0.591 ** (−2.78)
Variance sigma2 e	0.031 *** (19.92)	0.057 *** (19.35)	0.028 *** (19.85)
Wx			
lngdp	−0.173 (−1.29)	0.553 * (2.01)	0.075 (0.20)
lnge	0.021 (0.16)	0.222 (1.18)	0.651 ** (3.25)
lnis	0.074 (0.32)	−1.652 *** (−4.39)	−1.085 * (−2.32)
lnes	−0.023 (−0.20)	0.357 (1.66)	0.221 (1.10)
lnroad	0.242 (1.77)	0.741 *** (3.40)	0.318 (1.66)
lner	0.315 (1.77)	0.273 (3.40)	1.230 ** (1.66)
lnpd	−0.286 (−0.81)	−0.023 (−0.13)	0.032 (0.08)
lneip	−0.061 (−0.86)	−0.096 (−0.69)	−0.158 (−1.26)
R-squared	0.136	0.139	0.058
Number of OBs	798	798	798

Note: *, **, and *** denote significance at the 10%, 5%, and 1% significance levels, respectively. The T-statistics are given in brackets.

**Table 6:** Spatial effect decomposition of SDM model.

Variable	LR Direct		LR Indirect		LR Total	
	Coefficient	T-Statistic	Coefficient	T-Statistic	Coefficient	T-Statistic
lnpgdp	0.229 ***	4.76	−0.018	−0.07	0.210	0.86
lnge	−0.057 *	−2.33	0.453 ***	3.29	0.397 **	2.89
lnis	0.033	0.61	−0.738 *	−2.29	−0.706 *	−2.14
lnes	−0.074 ***	−3.31	0.183	1.32	0.109	0.79
lnroad	−0.022	−1.03	0.212	1.58	0.189	1.38
lner	−0.120 **	−2.99	0.868 **	3.11	0.748 **	2.68
lnpd	−0.011	−0.25	0.014	0.05	0.003	0.01
lneip	−0.027 *	−2.26	−0.094	−1.12	−0.121	−1.46

Note: *, **, and *** denote significance at the 10%, 5%, and 1% significance levels, respectively.

**Table 7:** Robustness test results after independent variable replacement.

Variable	LR Direct		LR Indirect		LR Total	
	Coefficient	T-Statistic	Coefficient	T-Statistic	Coefficient	T-Statistic
lnpgdp	0.225 ***	4.82	−0.017	−0.07	0.208	0.89
lnge	−0.057 *	−2.33	0.472 ***	3.47	0.415 **	3.07
lnis	−0.027	−0.59	0.757 **	2.88	0.730 **	2.72
lnes	−0.079 ***	−3.54	0.207	1.49	0.128	0.93
lnroad	−0.023	−1.04	0.231	1.73	0.208	1.53
lner	−0.114 **	−2.89	0.975 ***	3.52	0.861 **	3.12
lnpd	−0.009	−0.22	0.026	0.10	0.017	0.06
lneip	−0.027 *	−2.30	−0.097	−1.21	−0.125	−1.57

Note: *, **, and *** denote significance at the 10%, 5%, and 1% significance levels, respectively.

**Table 8:** Robustness test results after spatial weight matrix replacement.

Variable	LR Direct		LR Indirect		LR Total	
	Coefficient	T-Statistic	Coefficient	T-Statistic	Coefficient	T-Statistic
lnpgdp	0.227 ***	4.72	−0.028	−0.11	0.199	0.83
lnge	−0.058 *	−2.35	0.458 ***	3.34	0.400 **	2.93
lnis	0.036	0.66	−0.695 *	−2.20	−0.659 *	−2.05
lnes	−0.074 **	−3.28	0.183	1.36	0.109	0.81
lnroad	−0.021	−0.98	0.202	1.53	0.181	1.34
lner	−0.117 **	−2.92	0.910 **	3.26	0.793 **	2.84
lnpd	−0.012	−0.27	0.001	0.0	−0.011	−0.04
lneip	−0.027 *	−2.32	−0.096	−1.15	−0.123	−1.49

Note: *, **, and *** denote significance at the 10%, 5%, and 1% significance levels, respectively.

**Table 9:** Heterogeneity Test results of the SDM model.

Variable	The Upstream Area			The Midstream Area			The Downstream Area		
	LR Direct	LR Indirect	LR Total	LR Direct	LR Indirect	LR Total	LR Direct	LR Indirect	LR Total
lnpgdp	0.087 (0.89)	0.072 (0.14)	0.159 (0.30)	0.167 * (2.15)	−0.720 (−1.87)	−0.553 (−1.40)	0.266 * (2.56)	−1.213 ** (−3.14)	−0.947 * (−2.35)
lnge	−0.094 ** (−2.66)	−0.132 (−0.68)	−0.226 (−1.09)	−0.090 (−1.87)	−0.203 (−1.05)	−0.294 (−1.53)	−0.135 (−1.92)	−0.108 (−0.40)	−0.243 (−0.92)
lnis	0.003 (0.03)	−0.035 (−0.05)	−0.032 (−0.04)	−0.002 (−0.03)	−0.918 * (−2.51)	−0.921 * (−2.47)	−0.001 (−0.01)	−0.480 (−1.01)	−0.480 (−0.89)
lnes	−0.109 ** (−3.04)	−0.170 (−0.85)	−0.279 (−1.36)	−0.081 * (−2.12)	−0.123 (−0.68)	−0.203 (−1.10)	−0.082 (−1.55)	−0.011 (−0.09)	−0.093 (−0.74)
lnroad	−0.027 (−0.92)	0.126 (1.12)	0.099 (0.85)	0.027 (0.62)	0.012 (0.06)	0.039 (0.19)	−0.007 (−0.14)	−0.037 (−0.22)	−0.044 (−0.25)
lner	−0.201 ** (−2.66)	0.642 (1.52)	0.441 (1.00)	−0.097 (−1.66)	−0.045 (−0.14)	−0.141 (−0.44)	−0.273 * (−2.00)	0.399 (1.05)	0.127 (0.32)
lnpd	−0.117 (−1.61)	−0.136 (−0.47)	−0.253 (−0.83)	−0.012 (−0.17)	0.012 (0.04)	−0.001 (−0.01)	0.126 (1.19)	−0.159 (−0.36)	−0.033 (−0.07)
lneip	−0.022 (−1.53)	−0.068 (−1.02)	−0.090 (−1.39)	0.010 (0.37)	0.032 (0.23)	0.043 (0.29)	−0.113 * (−2.52)	0.217 (1.77)	0.105 (0.90)

Note: * and ** denote significance at the 10% and 5% significance levels, respectively. The T-statistics are given in brackets.

## Data Availability

All data generated or analyzed during this study are included in this article.
